# Integrated multi-omics analysis reveals key genes and metabolic regulatory networks involved in the drought stress response of potato

**DOI:** 10.3389/fpls.2026.1954199

**Published:** 2026-10-06

**Authors:** Yaqi Liu, Guga Suri, Dalai Wuyun, Ying Chu, Sujun Liu

**Affiliations:** 1 College of Life Science and Technology, Inner Mongolia Normal University, Hohhot, China; 2 Key Laboratory of Biodiversity Conservation and Sustainable Utilization in Mongolian Plateau, College of Life Science and Technology, Inner Mongolia Normal University, Hohhot, China

**Keywords:** drought stress, gene expression, metabolic pathways, metabolites, potato

## Abstract

**Introduction:**

Against the backdrop of global warming, drought events have become increasingly frequent and represent a major environmental stress factor limits potato yield stability and sustained yield improvement.

**Methods:**

In this study, integrated transcriptomics and widely targeted metabolomics were used to characterize the response of the potato cultivar ‘Jizhangshu 12’ to moderate (T1) and severe (T2) drought stress, and RT-qPCR was used to validate key genes.

**Results and discussion:**

1,135 differentially expressed genes and 39 differentially accumulated metabolites were identified under moderate drought stress, whereas 505 differentially expressed genes and 158 differentially accumulated metabolites were identified under severe drought stress. GO and KEGG enrichment analyses indicated that phenylpropanoid-flavonoid biosynthesis, α-linolenic acid metabolism, glycerophospholipid metabolism, and MAPK-hormone signaling pathways were core modules involved in the drought response of potato. The COMT1 gene LOC102601955, F3’H gene LOC102596126, ABCG transporter gene LOC102602737, and WRKY transcription factor gene LOC102587289 were identified as core candidate drought-tolerance genes. The MAPK pathway may positively regulate lipid and steroid accumulation while negatively regulating flavonoid and phenolic biosynthesis. These findings elucidate distinct transcriptional-metabolic regulatory networks and provide candidate genes and metabolites for breeding drought-tolerant potato cultivars.

## Introduction

With the progression of global climate change, drought stress has become one of the most common abiotic stress factors affecting plant growth and development, exerting severe adverse effects on global crop productivity and causing substantial economic losses (Ahuja et al., 2010; Buragohain et al., 2024). Potato (*Solanum tuberosum* L.), the fourth most important food crop worldwide, is widely cultivated in Asia and Africa (Li, 2008; Liang et al., 2018). Owing to its relatively shallow root system and limited capacity to absorb soil water from deeper layers, potato plants are highly susceptible to drought stress (Nasir and Toth, 2022). Therefore, a comprehensive investigation of drought-tolerance mechanisms in potato is of great significance for ensuring food security.

Plants respond to drought stress by modulating morphological traits, physiological functions, metabolic processes, and gene expression (Yan et al., 2021). Dynamic changes in metabolites are important outcomes of plant responses to external environmental stresses and the development of stress tolerance. The types and abundance of metabolites can reflect the physiological status of plants under stress conditions (Aliche et al., 2020; Wang et al., 2020). Numerous studies have demonstrated that drought stress induces the accumulation of phenolic acids (including caffeic acid, *p*-coumaric acid, and ferulic acid) in potato. Phenolic compounds and flavonoids alleviate oxidative damage by scavenging reactive oxygen species (ROS), representing one of the core defense mechanisms underlying potato drought response (Mutanda et al., 2026). Gao et al. found that drought-tolerant potato cultivars accumulated abundant flavonoid and phenolic-acid antioxidant metabolites in leaves, confirming that phenylpropanoid metabolism and flavonoid biosynthesis are core secondary-metabolic pathways in potato responding to drought stress (Gao et al., 2026). In terms of gene expression, studies have shown that drought stress affects the expression of genes involved in photosynthesis, signal transduction, lipid metabolism, and carbohydrate metabolism in potato (Chen et al., 2019). Previous studies have demonstrated that the MYB, WRKY, and NAC families play key roles in regulating drought tolerance in potato (Sun et al., 2019; Yan et al., 2021). Liu et al (Liu et al., 2018). revealed the effects of drought stress and rewatering on key enzymes and gene expression in the flavonoid biosynthesis pathway of potato. Aliche et al (Aliche et al., 2020)elucidated the mechanism of carbon partitioning in potato under drought stress.

Transcriptomics and metabolomics are two key high-throughput technologies that play critical roles in studies of plant stress tolerance (Du et al., 2025; Lv et al., 2025). By integrating transcriptomic and metabolomic data, key genes related to stress tolerance, important metabolic pathways, and regulatory networks can be identified, providing a theoretical basis for breeding stress-tolerant cultivars and understanding stress-tolerance mechanisms (Choudhary et al., 2025). In potato, drought-responsive genes and metabolites are enriched in starch and sucrose metabolism and plant hormone signal transduction pathways (Chen et al., 2019), and integrated transcriptomic and metabolomic analysis of grafted potato showed that flavonoids participate in drought adaptation through the MYB-WD40-bHLH complex (Jian et al., 2024).

‘Jizhangshu 12’ is a major potato cultivar grown in the arid and semi-arid regions of North China and in the irrigated areas of Northwest China. It exhibits strong adaptability, stable yield performance, and substantial regional cultivation value. Existing studies have shown that its drought-tolerance coefficient can reach 0.67 under drought stress; this cultivar maintains stable photosynthetic performance, root activity and antioxidant systems under drought conditions, and possesses excellent drought adaptability (Zhao et al., 2022). Existing studies on drought tolerance in this cultivar have mostly been limited to phenotypic evaluation, agronomic traits, or single-omics analyses, lacking a systematic mechanistic investigation integrating transcriptomics and metabolomics. The aims of this study were to integrate transcriptomic and widely targeted metabolomic approaches to characterize the coordinated transcriptional-metabolic responses of ‘Jizhangshu 12’ to moderate and severe drought stress, and to identify key genes, metabolites, and regulatory networks involved in drought tolerance. The findings of this study aim to enrich the molecular mechanism underlying drought responses in potato and provide a theoretical basis for molecular breeding of drought-tolerant potato and water-saving cultivation in arid regions.

## Materials and methods

### Experimental materials and design

Virus-free early-generation seed tubers (pre-basic seed tubers) of the potato cultivar ‘Jizhangshu 12’, provided by the Zhangjiakou Academy of Agricultural Sciences, Zhangjiakou, Hebei, China, were used in this study. The cultivar is adapted to arid and irrigated regions and is valued for its stable yield performance under limited water availability. A pot experiment was conducted under a rain-shelter facility at Hailiutu Science and Technology Park, Inner Mongolia Agricultural University. Each plastic pot, 30 cm in height and 20 cm in maximum diameter, contained 2.5 kg of sieved loam soil with a maximum field water-holding capacity of 22.13%. Uniform seed tubers weighing approximately 5 g were selected, and one tuber was planted in the center of each pot. After sowing, the soil was thoroughly watered and maintained at 65%–70% relative soil water content until the seedlings reached approximately 5 cm in height.

The experiment included three treatments: a control (CK) maintained at 65%–70% relative soil water content (RSWC); moderate drought stress (T1) maintained at 35%–40% RSWC; and severe drought stress (T2) maintained at 15%–20% RSWC. Each treatment had three independent biological replicates. Soil water content was controlled by weighing and watering daily between 9:00 and 10:00. Drought treatment was initiated when plant height reached approximately 5 cm and lasted for 14 days. On the final day of treatment, young leaves were collected from 10 individual plants per replicate and pooled evenly to form one bulked biological sample; therefore, each treatment was represented by three independent bulked biological samples (three biological replicates). All samples were immediately frozen in liquid nitrogen, stored at -80 °C, and subsequently subjected to transcriptome sequencing (RNA-seq approach) and metabolomic analysis. The same three biological replicates were used for RNA-seq, metabolomics, and RT-qPCR analyses.

### Transcriptomic analysis

Total RNA was extracted from freeze-dried and ground potato seedlings using a modified CTAB-PBIOZOL method (Chang et al., 1993). Briefly, approximately 100 mg of freeze-dried tissue powder was added to pre-warmed CTAB extraction buffer (2% (w/v) CTAB, 2% (w/v) PVP-40, 100 mM Tris-HCl (pH 8.0), 25 mM EDTA, 2 M NaCl, 0.5 g/L spermidine; 2% (v/v) β-mercaptoethanol was added immediately before use) at 65 °C, and incubated at 65 °C for 15 min with intermittent vortexing. An equal volume of chloroform-isoamyl alcohol (24:1, v/v) was added, followed by centrifugation at 12,000 × g for 10 min at 4 °C. After the aqueous phase was collected, PBIOZOL reagent (BioFlux, Hangzhou, China) was added, and RNA precipitation was performed with minor modifications according to the manufacturer’s instructions.

RNA concentration and purity were determined using a Qubit fluorometer (Thermo Fisher Scientific, USA), and RNA integrity was assessed with a Qsep400 biofragment analyzer (BiOptic Inc., New Taipei, Taiwan, China). Only RNA samples with RNA integrity number (RIN) ≥ 7.0 and A260/A280 absorbance ratios of 1.8–2.1were used for library construction and RT-qPCR validation. mRNA enrichment, library construction, and high-throughput paired-end 150 bp (PE150) sequencing on an Illumina NovaSeq 6000 platform were performed by Wuhan Maiwei Metabolic Biotechnology Co., Ltd. (Wuhan, China).

Raw sequencing reads were filtered using fastp to remove adapter sequences, reads with an N base content > 10%, and reads with low-quality bases (Q ≤ 20) accounting for > 50%, to obtain clean reads (Chen et al., 2018). A reference genome index was constructed using HISAT2, and clean reads were aligned to the potato reference genome. Gene expression abundance was quantified in fragments per kilobase of transcript per million mapped reads (FPKM) (Liao et al., 2014; Kim et al., 2015). Differentially expressed genes (DEGs) were identified using DESeq2, with screening thresholds set at adjusted P < 0.01 and |log_2_ fold change (log_2_FC)| > 1 (Love et al., 2014). GO functional enrichment analysis was performed using the clusterProfiler R package, and KEGG pathway enrichment was conducted using KOBAS combined with clusterProfiler (Ogata et al., 1999; Ashburner et al., 2000).

Ten functional marker genes were selected for RT-qPCR validation (**Supplementary Table S1**). Candidate genes were chosen from those showing significantly differential expression under both moderate and severe drought stress, with priority given to genes involved in core drought response pathways, including photosynthesis, cell wall pectin modification, phenylpropanoid transcriptional regulation, MAPK stress signaling, proteolysis and membrane lipid remodeling. Total RNA (1 μg) was used to synthesize first-strand cDNA using the PrimeScript RT reagent Kit with gDNA Eraser (Takara, Japan) according to the manufacturer’s instructions. The cDNA was diluted to 90 ng/μL with nuclease-free water to serve as the qPCR template. The qPCR reaction was prepared in a total volume of 25 μL, containing 12.5 μL TB Green Premix Ex Taq II, 1 μL each of forward and reverse primers, 2 μL cDNA template, and 8.5 μL ddH_2_O. Amplification was run on a CFX96 Touch real-time fluorescence quantitative PCR system (Bio-Rad, Hercules, CA, USA) with the following program: pre-denaturation at 95 °C for 30 s; 40 cycles of 95 °C for 5 s and 60 °C for 30 s; after amplification, melting curves were generated from 65 °C to 95 °C to verify primer specificity. Each sample was set with three technical and biological replicates. Glyceraldehyde-3-phosphate dehydrogenase (GAPDH, LOC102601955) was used as the reference gene, and the relative expression level of target genes was calculated using the 2^-ΔΔCT^ method (Livak and Schmittgen, 2001).

### Metabolomic analysis

Potato seedling samples were vacuum freeze-dried using a Scientz-100F freeze dryer, and subsequently ground to a fine powder using an MM 400 mixer mill (Retsch, Germany) at 30 Hz for 1.5 min. An aliquot of 50 mg lyophilized powder was accurately weighed and extracted with 1200 μL of pre-chilled 70% aqueous methanol (−20 °C) containing 1 μg/mL 2-chloro-L-phenylalanine as an internal standard for quantification (Sawada et al., 2009). Samples were subjected to intermittent vortex extraction, with vortexing conducted every 30 minutes for 30 seconds per cycle, for a total of 6 cycles, the mixture was centrifuged at 12–000 rpm and 4 °C for 3 min. The supernatant was filtered through a 0.22 μm organic-phase microporous membrane and transferred to an autosampler vial for UPLC-MS/MS analysis. Quality control (QC) samples were prepared by pooling equal volumes of supernatant from all individual samples to assess the stability of instrumental detection.

Chromatographic separation was performed on an ExionLC™ AD ultra-high performance liquid chromatography system (SCIEX, USA) equipped with an Agilent SB-C18 column (1.8 micrometers, 2.1 mm × 100 mm). The column oven temperature was maintained at 40 °C, the flow rate was set at 0.35 mL/min, and the injection volume was 2 μL. The mobile phase consisted of 0.1% formic acid in water (phase A) and 0.1% formic acid in acetonitrile (phase B). The gradient elution program was as follows: 0–9.00 min, linear gradient from 5% to 95% B; 9.00–10.00 min, hold at 95% B; 10.00–11.10 min, ramp down to 5% B; 11.10–14.00 min, re-equilibrate at 5% B. Methanol, acetonitrile (Merck, Germany) and formic acid (Aladdin, China) were all of chromatographic grade.

Mass spectrometric detection was carried out on a TripleTOF 6600 system (SCIEX, Canada) coupled with an electrospray ionization (ESI) source. The ion source temperature was set at 500 °C, and the spray voltages were 5500 V for positive ion mode and −4500 V for negative ion mode. The curtain gas, nebulizer gas and auxiliary gas were set at 25 psi, 50 psi and 60 psi, respectively, with nitrogen used as the collision gas. Data were acquired in multiple reaction monitoring (MRM) mode; declustering potential and collision energy were optimized to obtain characteristic ion pairs for each metabolite (Sawada et al., 2009). One QC sample was injected every 10 analytical runs to monitor data quality. Qualitative and quantitative identification of metabolites was performed based on the database developed by Wuhan Maiwei Metabolic Biotechnology Co., Ltd. (Wuhan, China). Chromatographic peak areas were used for relative quantification, and peak integration was corrected according to a previously established method (Fraga et al., 2010).

Differentially accumulated metabolites (DAMs) were screened by combining univariate and multivariate statistical analyses. Principal component analysis (PCA) and orthogonal partial least squares discriminant analysis (OPLS-DA) were performed as described previously (Nemeth, 2003), with screening thresholds set at variable importance in projection (VIP) > 1 and |log_2_ fold change (log_2_FC)| ≥ 1 (Thévenot et al., 2015). Pearson correlation coefficients were calculated to evaluate the biological reproducibility of samples (Chen et al., 2009). KEGG pathway annotation and enrichment analysis of DAMs were conducted, and statistical significance was determined by hypergeometric test (Ogata et al., 1999).

### Integrated multi-omics analysis

Differentially expressed genes (DEGs) and differentially accumulated metabolites (DAMs) were simultaneously mapped to the Kyoto Encyclopedia of Genes and Genomes (KEGG) pathway database to identify metabolic pathways co-enriched in both omics datasets and to elucidate the regulatory associations between gene expression and metabolite accumulation (Ogata et al., 1999).

Pearson correlation analysis was performed based on gene expression levels and metabolite abundances across all samples. Significant gene–metabolite association pairs were screened using thresholds of |r| ≥ 0.80 and P < 0.05.

Canonical correlation analysis (CCA) was used to explore potential linear relationships between the two sets of variables. Canonical variable pairs were constructed to characterize the overall correlation between DEGs and DAMs, thereby revealing the coordinated response patterns of the transcriptome and metabolome at a global level (Hotelling, 1992).

## Results and analysis

### Metabolomic and DAM analysis

#### Multivariate statistical analysis of metabolome

To characterize the global metabolic variation patterns of potato cultivar ‘Jizhangshu 12’ under different drought stress treatments, multivariate statistical analysis based on widely targeted metabolomics was performed. Principal component analysis (PCA) showed clear separation among control, moderate drought and severe drought samples, with biological replicates of each treatment clustering well, indicating that drought stress significantly altered the leaf metabolic profiles of potato (**Figures 1A, B**). Supervised OPLS-DA analysis was further applied to amplify inter-group metabolic differences, and complete discrimination was achieved between CK vs. T1 and CK vs. T2 groups (**Figures 1C, D**).

**Figure 1:**
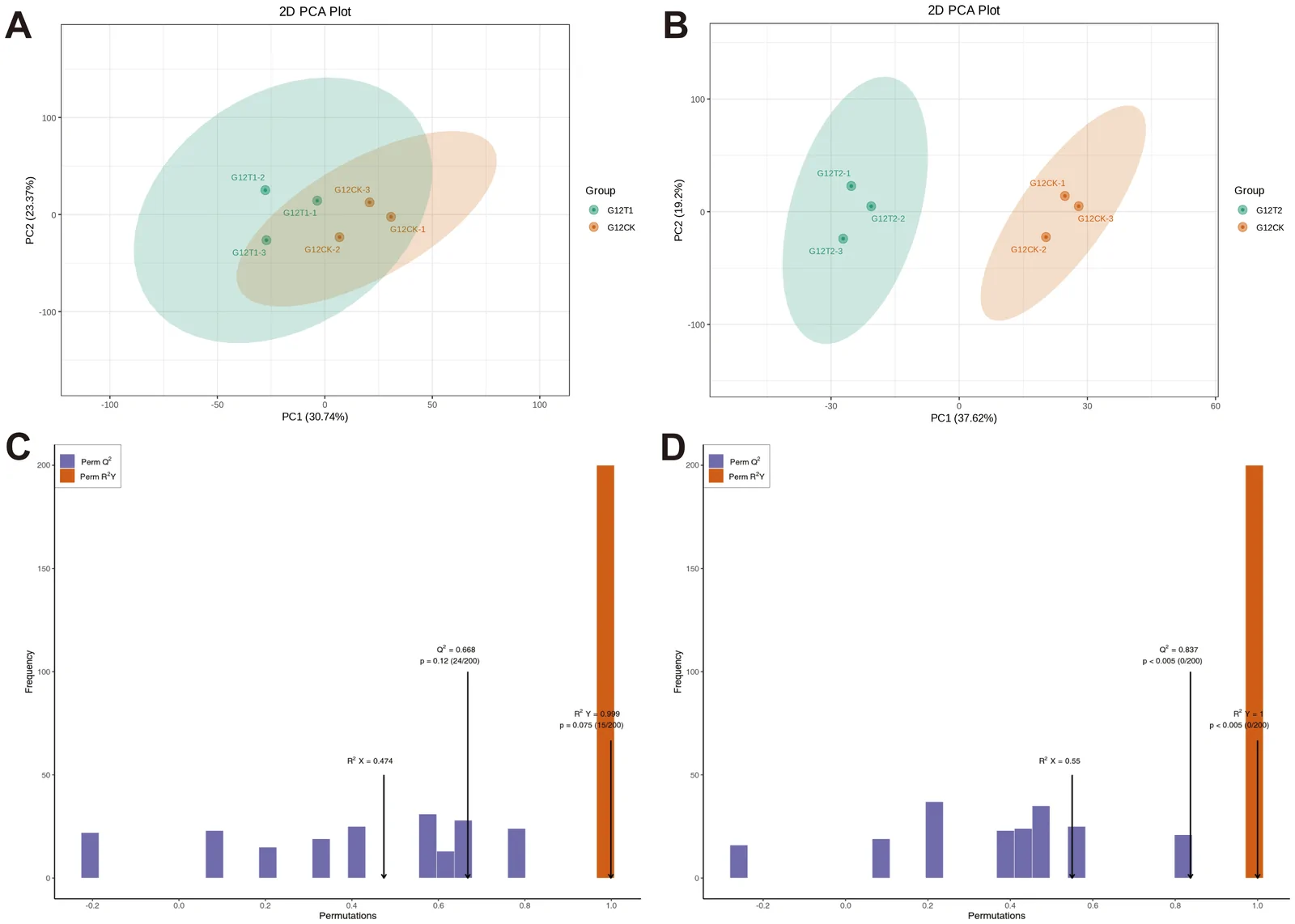
water-saving statistical analysis of metabolome under drought stress. **(A)** PCA score plot of T1 vs CK; **(B)** PCA score plot of T2 vs CK. Ellipses represent the 95% confidence interval. **(C)** OPLS DA score plot of T1 vs CK; **(D)** OPLS DA score plot of T2 vs CK. Different colors represent different treatment groups.

To avoid model overfitting, 200 permutation tests were conducted, and the results verified the validity of the OPLS-DA model with no overfitting (**Supplementary Figures S1A, B**). The reproducibility of transcriptome samples was evaluated using Pearson correlation coefficients between biological replicates. The sample correlation heatmap revealed high intra-group replicate correlation, confirming the reliable quality of sequencing samples (**Supplementary Figure S1**).

#### Effects of different drought stress treatments on the number of differential metabolites in potato

Significantly differentially accumulated metabolites were screened based on the criteria of |log2FC| ≥ 1 and P < 0.05, and volcano plots were generated (**Figure 2**). The results showed that 39 significantly different metabolites were identified under moderate drought stress (T1 vs CK), including 20 upregulated and 19 downregulated metabolites. Under severe drought stress (T2 vs CK), 158 significantly different metabolites were identified, including 67 upregulated and 91 downregulated metabolites. As drought stress intensified, the total number of differential metabolites increased markedly, and the proportion of downregulated metabolites also increased significantly. These results indicate that severe drought caused stronger disturbances in the plant metabolome and more extensive inhibition of primary metabolism.

**Figure 2:**
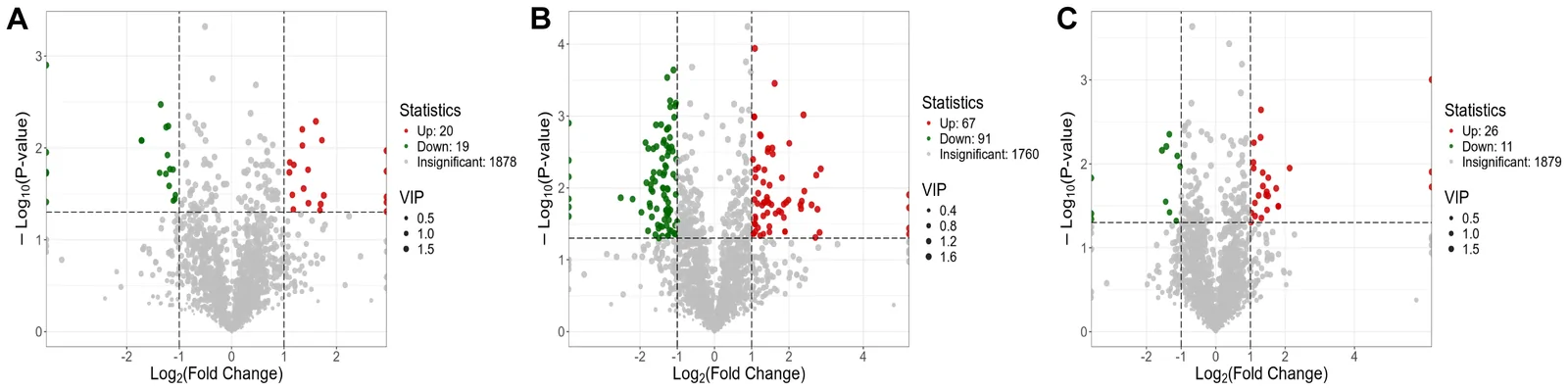
Volcano plots of differential metabolites under different drought stress treatments. **(A)** T1 vs CK. **(B)** T2 vs CK. **(C)** T2 vs T1. The x-axis is log2(fold change) and the y-axis is -log10(P value). Red and green points represent significantly up- and down-regulated metabolites (|log2FC| ≥ 1 and P < 0.05), respectively.

#### Effects of different drought stress treatments on metabolic pathways of differential metabolites in potato

Metabolites with significantly different abundances were mapped to the KEGG database, and pathway enrichment analysis was performed to identify the major pathways involved in the differential metabolites under different drought conditions in potato (**Figure 3**). Compared with the control group, differential metabolites under moderate drought stress were significantly enriched in pathways related to biosynthesis of secondary metabolites, phenylpropanoid biosynthesis, biosynthesis of amino acids, flavonoid biosynthesis, and arginine biosynthesis (**Figure 3A**). Compared with the control, differential metabolites under severe drought stress were significantly enriched in linoleic acid metabolism, α-linolenic acid metabolism, tryptophan metabolism, and lysine biosynthesis pathways (**Figure 3B**).

**Figure 3:**
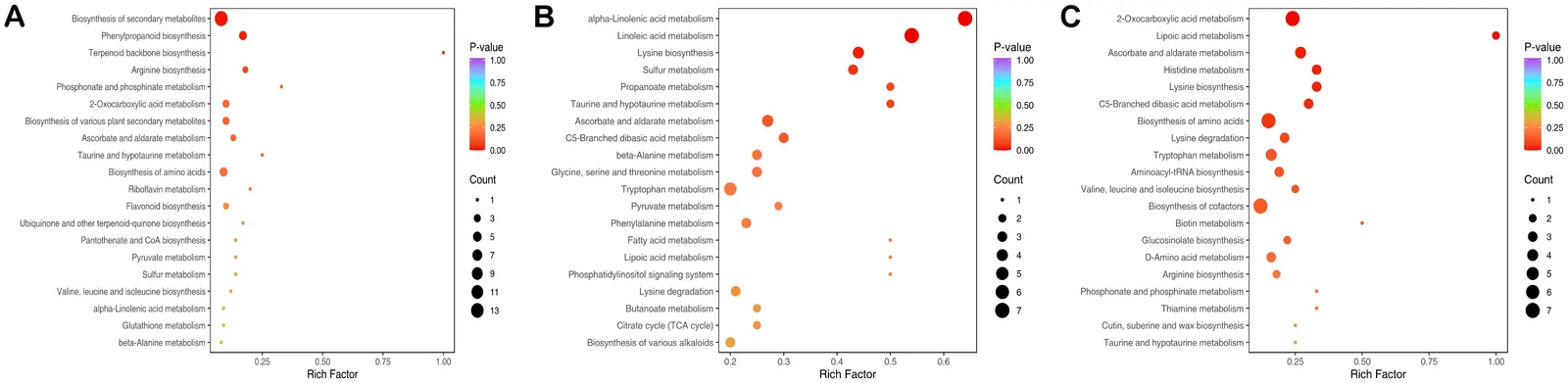
KEGG pathway enrichment bubble plots of differential metabolites under different drought stress treatments. **(A)** T1 vs CK. **(B)** T2 vs CK. **(C)** T2 vs T1.

#### Effects of different drought stress treatments on the classes of differential metabolites in potato

Based on the KEGG pathway enrichment results, metabolic pathways with high enrichment significance were selected, and hierarchical clustering analysis was performed for all differential metabolites within these pathways (**Figure 4**). The clustering results showed that, under moderate drought stress, the differential metabolites included multiple classes, such as amino acids and their derivatives, organic acids, flavonoids, phenolic acids, lignans and coumarins, and lipids. Both upregulated and downregulated members were observed within each class, indicating a bidirectional regulatory response involving multiple pathways (**Figure 4A**).

**Figure 4:**
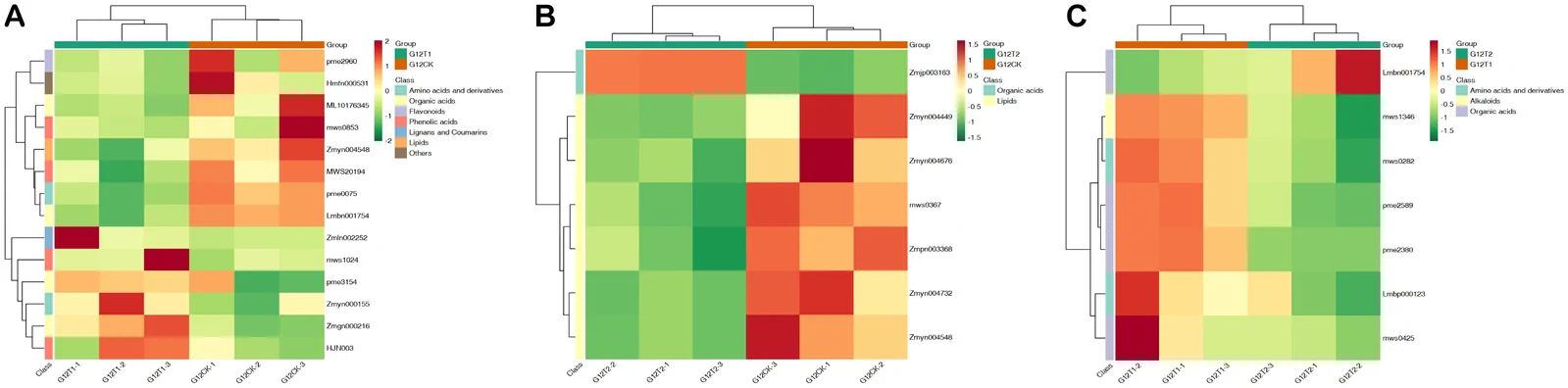
Hierarchical clustering heatmaps of differential metabolites under different drought stress treatments. **(A)** T1 vs CK. **(B)** T2 vs CK. **(C)** T2 vs T1.

Under severe drought stress, the differential metabolites were mainly concentrated in two major classes: organic acids and lipids. Organic acids showed stress-specific accumulation, whereas lipid metabolites exhibited an overall significant downward trend (**Figure 4B**). Compared with the control, among the top 20 significantly differential metabolites under moderate and severe drought treatments (**Figure 5**), seven metabolites were commonly upregulated, including steroidal saponins/steroidal glycosides, flavonoids, and alkaloids, whereas three metabolites were commonly downregulated, including phenolamides, sesquiterpene lactones, and depsides.

**Figure 5:**
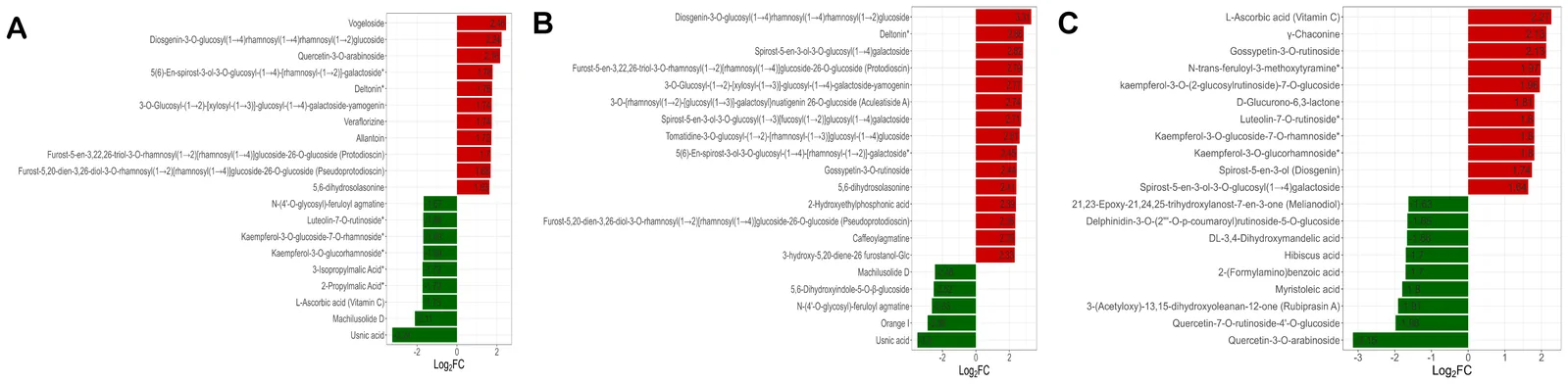
Fold changes of the top 20 significantly differential metabolites in potato cultivar ‘Jizhangshu 12’ under different drought stress treatments. **(A)** T1 vs CK. **(B)** T2 vs CK. **(C)** T2 vs T1. Note: The x-axis represents the log2FC values of differential metabolites, that is, the base-2 logarithm of fold change, and the y-axis represents the differential metabolites. Red indicates upregulated metabolite content, whereas green indicates downregulated metabolite content.

Overall, metabolite accumulation in potato differed under drought stress, which may be associated with regulatory mechanisms involved in potato drought tolerance.

### Quality control analysis of transcriptomic data

#### Effects of different drought stress treatments on the number of differentially expressed genes in potato

Transcriptome sequencing (RNA-seq approach) was performed for all potato leaf samples. Q20 > 98% and Q30 > 94% were observed across all samples, indicating high-quality sequencing data (**Supplementary Table S2**). Clean reads were used for subsequent gene expression analysis.

The number of differentially expressed genes (DEGs) among different treatments is shown in **Figure 6**. Compared with CK, 1,135 genes were significantly differentially expressed under the T1 treatment, including 199 upregulated and 936 downregulated genes. Under the T2 treatment, 505 genes were significantly differentially expressed, including 181 upregulated and 324 downregulated genes. These results indicate that downregulated genes predominated under both drought stress treatments. Compared with CK, the T1 treatment induced a higher total number of DEGs and a greater number of downregulated genes.

**Figure 6:**
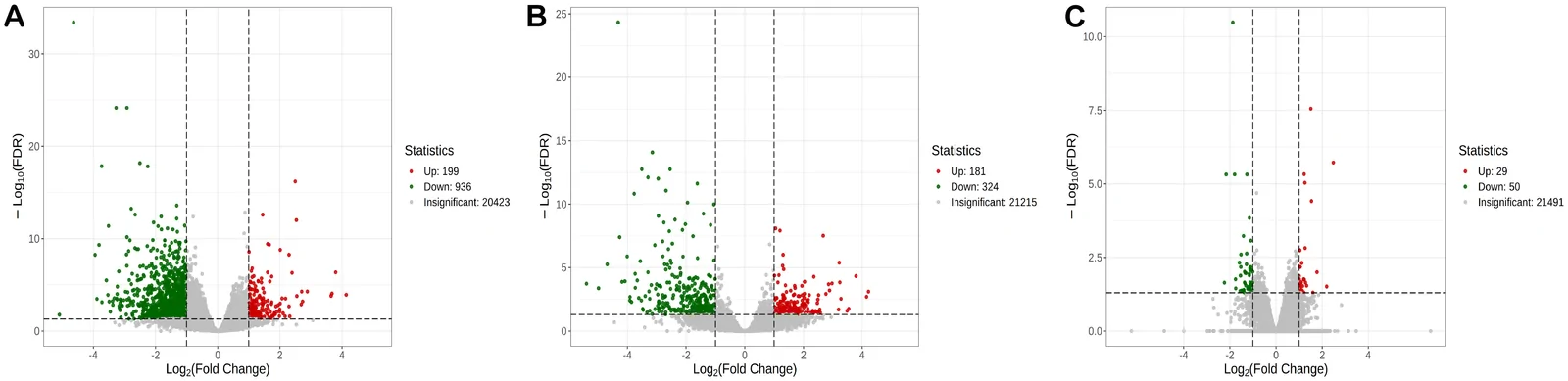
Volcano plot of differentially expressed genes among different treatments. **(A)** T1 vs CK. **(B)** T2 vs CK. **(C)** T2 vs T1. The x−axis is log_2_(fold change) and the y−axis is -log_1__0_(FDR). Red and green points represent significantly up− and down−regulated genes (|log_2_FC| ≥ 1 and FDR < 0.05), respectively.

#### Effects of different drought stress treatments on metabolic pathways of differentially expressed genes in potato

Differentially expressed genes in potato cultivar ‘Jizhangshu 12’ under different drought stress treatments were mapped to the KEGG database to identify the major pathways in which these genes were involved (**Figure 7**). Compared with CK, the significantly enriched pathways under the T1 treatment included metabolic pathways, photosynthesis–antenna proteins, biosynthesis of secondary metabolites, pentose and glucuronate interconversions, starch and sucrose metabolism, DNA replication, glycosphingolipid biosynthesis–ganglion series, cutin, suberin and wax biosynthesis, and glycerophospholipid metabolism (**Figure 7A**).

**Figure 7:**
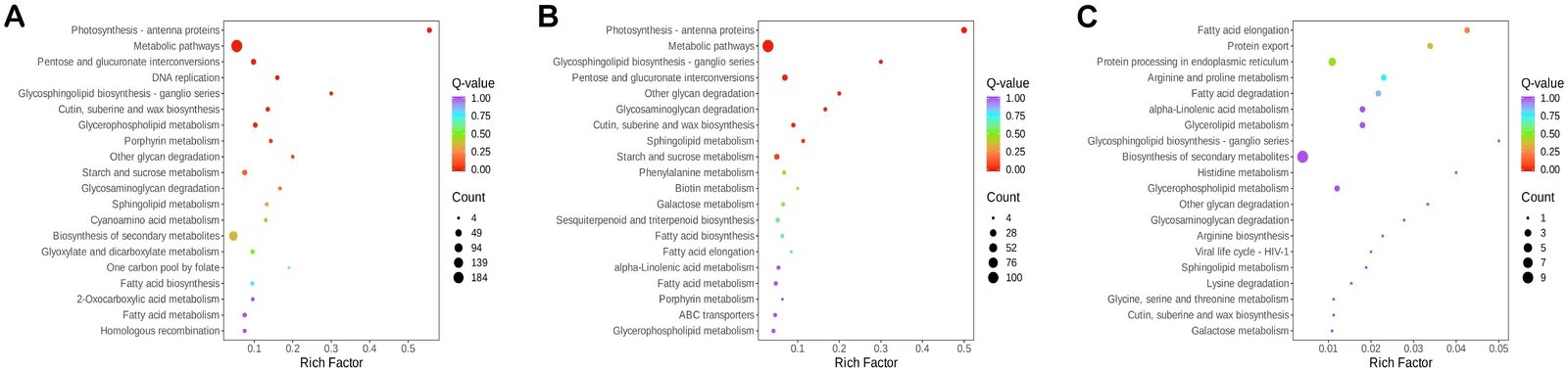
KEGG enrichment scatter plot of differentially expressed genes among different treatment. **(A)** T1 vs CK. **(B)** T2 vs CK. **(C)** T2 vs T1. Note: The y-axis represents the names of KEGG pathways, and the x-axis represents the enrichment factor of DEGs annotated to each pathway.

Compared with CK, the significantly enriched pathways under the T2 treatment included metabolic pathways, photosynthesis-antenna proteins, glycosphingolipid biosynthesis-ganglion series, pentose and glucuronate interconversions, starch and sucrose metabolism, and cutin, suberin and wax biosynthesis (**Figure 7B**). Compared with CK, seven additional pathways were enriched under the T2 treatment, including phenylalanine metabolism, biotin metabolism, galactose metabolism, sesquiterpenoid and triterpenoid biosynthesis, fatty acid elongation, α-linolenic acid metabolism, and ABC transporters.

#### Effects of different drought stress treatments on functional GO terms of differentially expressed genes in potato

Differentially expressed genes were mapped to the Gene Ontology (GO) database, and GO functional annotation and enrichment analysis were performed for DEGs in potato cultivar ‘Jizhangshu 12’ under different drought stress treatments (**Figure 8**). Compared with CK, the GO terms with the highest proportions of DEGs under the T1 treatment were as follows: biological processes, including cellular polysaccharide metabolic process and carbohydrate catabolic process; cellular components, including apoplast, anchored component of membrane, and protein-DNA complex; and molecular functions, including hydrolase activity acting on O-glycosyl compounds, enzyme inhibitor activity, and glucosyltransferase activity (**Figure 8A**).

**Figure 8:**
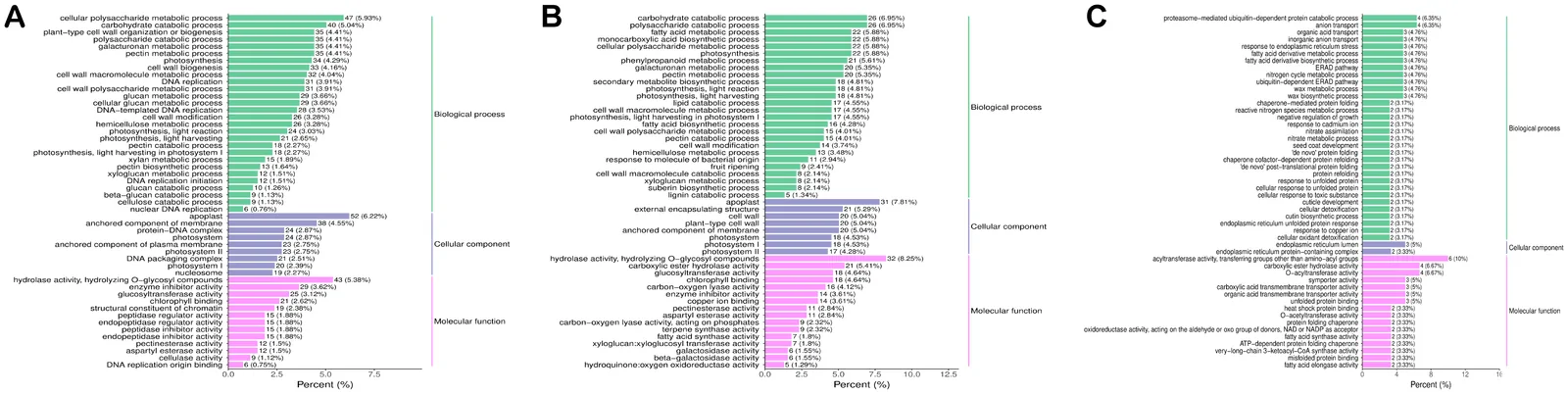
GO enrichment bar chart of differentially expressed genes among different treatments. **(A)** T1 vs CK. **(B)** T2 vs CK. **(C)** T2 vs T1. Note: The y-axis represents the names of GO terms, and the x-axis represents the number of DEGs annotated to each term and their proportion relative to the total annotated DEGs.

Compared with CK, the GO terms with the highest proportions of DEGs under the T2 treatment were as follows: biological processes, including carbohydrate catabolic process, polysaccharide catabolic process, and fatty acid metabolic process; cellular components, including apoplast, external encapsulating structure, and cell wall; and molecular functions, including hydrolase activity acting on O-glycosyl compounds, carboxylic ester hydrolase activity, and glucosyltransferase activity (**Figure 8B**).

Under different drought stress treatments, biological process terms contained the largest number of GO entries and the highest proportion of DEGs in potato cultivar ‘Jizhangshu 12’, indicating that these genes were mainly involved in dynamic physiological regulation. Compared with CK, the T2 treatment showed 25 newly enriched terms, including fatty acid metabolic process, phenylpropanoid metabolic process, secondary metabolite biosynthetic process, suberin biosynthetic process, external encapsulating structure, and photosystem II.

#### Validation and analysis of quantitative real-time PCR results

To verify the reliability of the transcriptome sequencing (RNA-seq approach) results, we performed RT-qPCR expression validation on 10 screened drought-responsive differentially expressed genes. Detailed information on the corresponding specific primers is provided in **Supplementary Table S1**.

The results showed that the expression trends of the validated genes under different drought stress treatments detected by RT-qPCR were highly consistent with the FPKM expression profiles from the transcriptome (**Figure 9**), and the correlation between the two datasets was statistically significant (P < 0.01). The favorable overall concordance of gene expression patterns indicates that the transcriptome sequencing(RNA-seq) data obtained in this study are reliable, and the differentially expressed genes identified herein may exert important regulatory roles in the drought response of potato.

**Figure 9:**
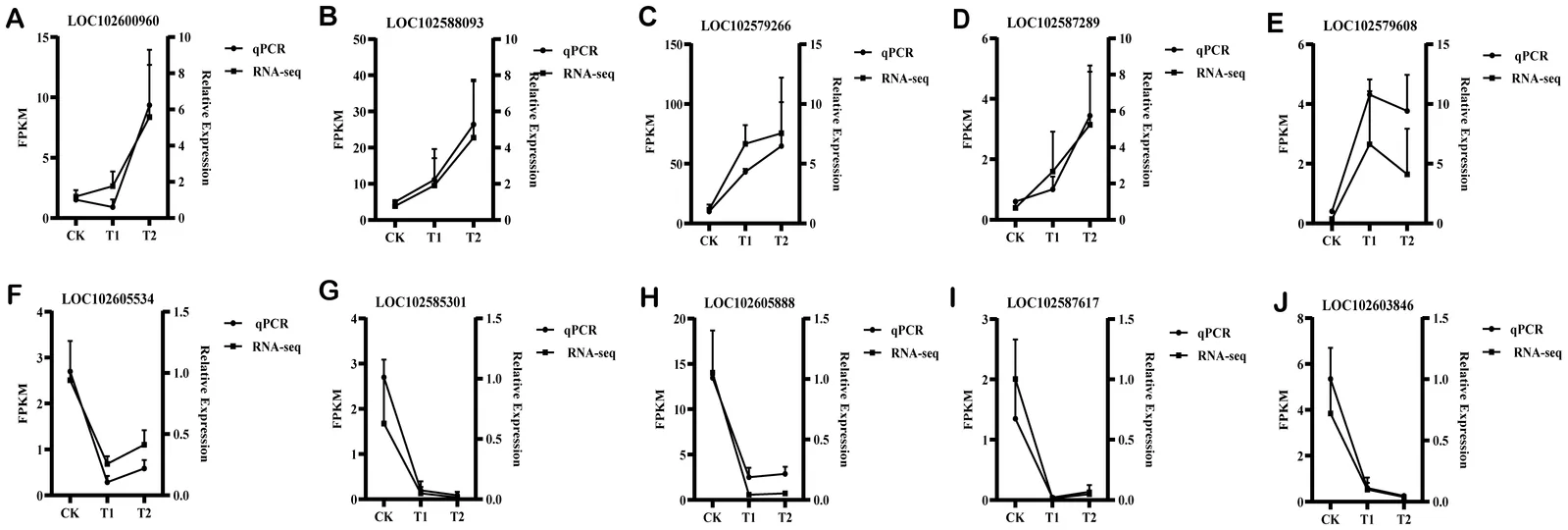
RT-qPCR validation of gene expression profiles obtained from RNA-seq. **(A–J)** Expression patterns of ten candidate genes under control (CK), moderate drought (T1), and severe drought (T2) treatments. The left y axis represents gene expression level (FPKM) from RNA-seq data; the right y-axis indicates relative expression determined by RT-qPCR. Error bars represent standard deviation (SD) of three biological replicates. Black circles correspond to RNAseq results, and black diamonds represent RT-qPCR results.

### Integrated transcriptomic and metabolomic analysis

#### Association characteristics between pathway gene sets and metabolite classes in potato under different drought stress conditions

To elucidate the coordinated transcriptional and metabolic responses of potato leaves under different drought intensities, Pearson correlation analysis was performed between all differentially expressed genes and differentially accumulated metabolites in the moderate and severe drought groups. Significant associations were screened using the criteria of |r| ≥ 0.8 and P < 0.05, and a correlation heatmap was generated (**Figure 10**). The results showed that, under both stress gradients, gene sets involved in seven core pathways, including hormone signal transduction, MAPK signaling, photosynthesis, starch and sucrose metabolism, phenylpropanoid biosynthesis, plant–pathogen interaction, and pentose and glucuronate interconversions, were significantly positively correlated with lipids and steroid metabolites. In contrast, all pathway gene sets were significantly negatively correlated with secondary metabolites, including flavonoids, phenolics, and alkaloids.

**Figure 10:**
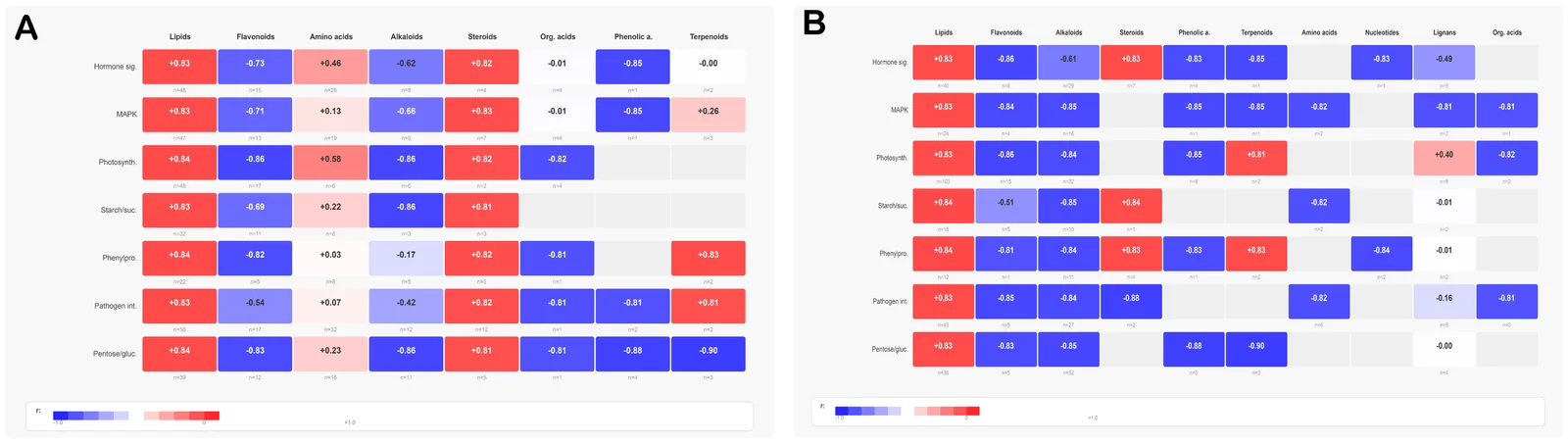
Correlation heatmaps between pathway gene sets and metabolite classes under different drought stress treatments. **(A)** T1 vs CK. **(B)** T2 vs CK.

The coordinated patterns of secondary metabolism differed markedly between the two drought intensities. Under moderate drought stress, the MAPK signaling, phenylpropanoid biosynthesis, and plant–pathogen interaction pathways were significantly positively correlated with terpenoids. The starch and sucrose metabolism pathway showed no significant antagonistic relationship with flavonoids, and amino acids exhibited only a weak positive correlation. Under severe drought stress, only the photosynthesis pathway maintained a positive correlation with terpenoids, whereas all pathways showed strong negative correlations with flavonoids. In addition, only the MAPK pathway was accompanied by the coordinated upregulation of amino acids, indicating that the number of pathways involved in diverting carbon skeletons toward secondary defensive metabolites was markedly reduced. It should be noted that this heatmap reflects only the overall trends between pathway gene sets and metabolite classes, and the expression patterns of individual metabolites within the same class may differ from the overall trend.

#### Co-enrichment characteristics of transcriptomic and metabolic pathways in potato under different drought stress conditions

Based on integrated transcriptomic and metabolomic analyses, we performed KEGG pathway co-enrichment analysis of differentially expressed genes (DEGs) and differentially accumulated metabolites (DAMs) in potato ‘Jizhangshu 12’ under different drought stress treatments (**Figure 11**). Compared with the control (CK), a total of 6 pathways were significantly co-enriched with both DEGs and DAMs in the T1 treatment (**Figure 11A**), namely phenylpropanoid biosynthesis, flavonoid biosynthesis, amino acid biosynthesis, pyruvate metabolism, 2-oxocarboxylic acid metabolism, and ascorbate and aldarate metabolism. Compared with the control (CK), a total of 6 pathways were significantly co-enriched with both DEGs and DAMs in the T2 treatment (**Figure 11B**), namely pentose and glucuronate interconversions, α-linolenic acid metabolism, ABC transporters, phenylpropanoid biosynthesis, fatty acid metabolism, and glycerophospholipid metabolism.

**Figure 11:**
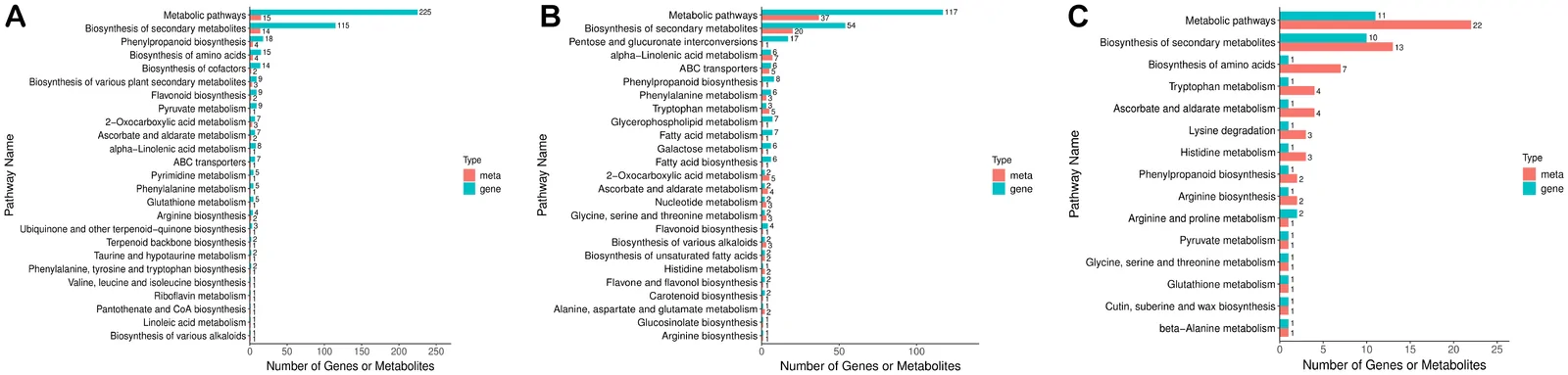
Statistical bar charts of KEGG pathway enrichment for differentially expressed genes and differentially accumulated metabolites under different drought stress treatments. **(A)** T1 vs CK. **(B)** T2 vs CK. **(C)** T2 vs T1.

#### Regulatory network analysis of differentially expressed genes and differentially accumulated metabolites in core drought-tolerance pathways

Based on the KEGG gene-metabolite co-enrichment results under moderate drought stress (**Figure 11A**), differentially expressed genes from six core drought-tolerance pathways were extracted and subjected to Pearson correlation analysis with differentially accumulated metabolites under the corresponding stress condition. Using |r| ≥ 0.8 and P < 0.05 as screening thresholds, a gene-metabolite regulatory network under moderate drought stress was constructed (**Supplementary Figure S3A**). The results showed that a total of 70 significant association pairs were identified, involving 34 differentially expressed genes and 15 differentially accumulated metabolites. In this network, LOC102601955 (*COMT1*) in the phenylpropanoid biosynthesis pathway was significantly and positively correlated with membrane protective components and terpenoid antioxidant metabolites; LOC102596126 (*F3’H*) in the flavonoid biosynthesis pathway was negatively correlated with flavonoid metabolites and participated in flavonoid metabolism remodeling. In the amino acid biosynthesis pathway, LOC102595103 (*GS1-1*) was positively correlated with osmoprotective metabolites, whereas LOC102603300 (*NADP-ME*) was negatively correlated, jointly regulating the homeostasis of amino acid metabolism. In the pyruvate metabolism pathway, LOC102597674 (*PKG*) was positively correlated with steroidal secondary metabolites; the key gene LOC102601823 (*mMDH*) was significantly and positively correlated with multiple organic acids and was involved in maintaining carbon metabolism homeostasis. In addition, LOC102597684 (*APX3*) in the ascorbate and aldarate metabolism pathway was positively correlated with membrane lipid components and negatively correlated with flavonoids, participating in the regulation of oxidative balance.

Based on the KEGG gene-metabolite co-enrichment results under severe drought stress (**Figure 11B**), all differentially expressed genes within six core drought-tolerance pathways were extracted and subjected to Pearson correlation analysis with all differentially accumulated metabolites under the same stress condition. Using |r| ≥ 0.8 and *P* < 0.05 as the screening thresholds, a gene-metabolite regulatory network under severe drought stress was constructed (**Supplementary Figure S3B**). This analysis yielded a total of 129 significant association pairs consisting of 20 differentially expressed genes and 31 differentially accumulated metabolites. In this network, LOC102586126, LOC102578151 (*LACS4*) and LOC102590505 (*KAS I)* from the glycerophospholipid and fatty acid metabolism pathway, together with LOC102602737 (*ABCG11)* from the ABC transporter pathway, showed significant correlations with multiple membrane lipid metabolites. Gene family members encoding pectate lyase and pectinesterase from the pentose and glucuronate interconversions pathway, as well as the hub genes LOC102601955 (*COMT1*) and LOC102595175 from the phenylpropanoid biosynthesis pathway, were also involved in this association network. Among them, *COMT1* was positively correlated with terpenoids, steroidal glycosides and membrane lipid metabolites, but negatively correlated with phenolamide derivatives; LOC102595175 also showed a negative correlation with phenolamide derivatives.

Detailed information on the key drought-responsive genes and differentially accumulated metabolites screened by integrated transcriptomic and metabolomic analysis is presented in **Supplementary Tables S3**, **S4**. The raw sequencing data have been deposited in a public database.

## Discussion

### Coordinated regulatory mechanism of phenylpropanoid-flavonoid and amino acid-antioxidant systems under moderate drought stress

Drought stress disrupts plant metabolic homeostasis and reshapes the accumulation patterns of secondary metabolites (Wang et al., 2025). The accumulation of flavonoids in plant tissues can serve as a characteristic marker of stress exposure. Their abundant phenolic hydroxyl groups can efficiently scavenge drought-induced reactive oxygen species (ROS), alleviate oxidative damage, and enhance plant drought tolerance (Laoué et al., 2022). Under drought stress, sweet potato accumulates large amounts of flavonoids, thereby improving drought tolerance (Yin et al., 2025). The results of this study showed that moderate and severe drought stress promoted the synthesis and accumulation of quercetin-3-O-arabinoside and gossypetin-3-O-rutinoside, respectively.

Phenylpropanoid biosynthesis is a core secondary metabolic pathway involved in plant drought responses, and its downstream branches include multiple stress-tolerance-related metabolites, such as flavonoids, phenolic acids, and coumarins (Feng et al., 2024). The hub gene in this pathway, LOC102601955, encodes caffeic acid 3-methyltransferase (*COMT1*), a key methyltransferase for lignin monomer biosynthesis (Saluja et al., 2021). Correlation analysis showed that this gene was significantly and positively correlated with membrane lipid components and terpenoid antioxidant metabolites. Previous studies have demonstrated that reduced COMT activity inhibits lateral root formation and impairs plant water absorption capacity under drought conditions (Saluja et al., 2021); in walnut, COMT improves plant drought tolerance by regulating lignin biosynthesis and the antioxidant system (Ma et al., 2024). Combined with our results, we speculate that *COMT1* not only participates in cell wall lignification, but also coordinately regulates the accumulation of membrane lipids and terpenoid protective metabolites, jointly mediating stress adaptive regulation in potato under moderate drought.

The flavonoid biosynthesis pathway is an essential component of the antioxidant defense system in potato under moderate drought stress. The key gene LOC102596126 in this pathway encodes flavonoid 3’-monooxygenase (*F3’H*), a cytochrome P450 superfamily member that catalyzes 3’-hydroxylation of the flavonoid B-ring and is critical for the biosynthesis of highly antioxidant flavonoid derivatives such as quercetin (Shi, 2006; Park et al., 2021). In this study, *F3’H* expression was significantly and negatively correlated with isoorientin, a final flavonoid product. Previous studies have demonstrated that F3’H is widely involved in plant drought stress responses: in tobacco, drought-induced F3’H mediates abiotic stress tolerance by modulating flavonoid profiles (Guo et al., 2025); in sweet orange, the *CsF3’H* gene is significantly upregulated under drought stress, and its overexpression in *Arabidopsis* reduces reactive oxygen species (ROS) levels and enhances antioxidant capacity and drought tolerance (Guo et al., 2011).

In the amino acid biosynthesis pathway, LOC102595103 encodes cytosolic glutamine synthetase (*GS1-1*), which promotes the accumulation of osmoprotectants. This isozyme is predominantly localized in the potato cytoplasm and significantly upregulated under drought stress (Rajkumar et al., 2026). Ascorbate and aldarate metabolism acts as a core antioxidant pathway for reactive oxygen species (ROS) scavenging in plants (Hasanuzzaman et al., 2020), in which LOC102597684 encodes peroxisomal ascorbate peroxidase *APX3*, a key functional gene for ROS clearance (Li et al., 2023). Previous studies have demonstrated that the *APX3* gene in alfalfa is upregulated by drought, and alleviates drought-induced oxidative damage via H_2_O_2_ scavenging (Chen et al., 2024). Correlation analysis in this study showed that this gene was positively correlated with membrane lipid components and negatively correlated with flavonoids, indicating that the antioxidant system coordinately regulates membrane lipid and flavonoid metabolism and is jointly involved in adaptive regulation under drought stress.

### Lipid metabolism remodeling and energy metabolism shift under severe drought stress

Lipids are important structural and functional components of membranes. Under drought stress, plants can obtain energy through lipid degradation, while lipid-derived components, such as waxes, cuticle, and lignin, help alleviate drought stress and tissue damage (Gaude et al., 2008; Samuels et al., 2008). Zi et al. found that the α-linolenic acid metabolic pathway was significantly enhanced in maize seedlings under drought stress. Maize adjusts fatty acid abundance under drought stress to maintain membrane integrity and activate pathways suitable for lipid metabolism (Zi et al., 2022). Studies have shown that cultivars with stronger drought tolerance often exhibit more active glycerophospholipid metabolism, which enables rapid membrane lipid turnover to remove oxidatively damaged fatty acid chains and reincorporate healthy fatty acids (Perlikowski et al., 2022).

Under severe drought stress, the glycerophospholipid metabolism, fatty acid metabolism and ABC transporter pathways are significantly enriched, and jointly mediate the stress-adaptive remodeling of the membrane system in potato. In this study, LOC102586126, LOC102578151 (*LACS4*), LOC102590505 (*KAS I*) and the ABC transporter family gene LOC102602737 (*ABCG11*) were significantly differentially expressed, which are involved in membrane phospholipid hydrolysis, fatty acid activation, *de novo* fatty acid synthesis in chloroplasts, and transmembrane transport of lipids and cuticular precursors, respectively (Wu and Xue, 2010; Liang et al., 2023). These genes collectively promote the accumulation of unsaturated lysophosphatidylcholines and lysophosphatidylethanolamines, and maintain the structural stability of cell membranes under drought stress. Previous studies have shown that phospholipase A regulates triacylglycerol biosynthesis in leaves and participates in drought response in soybean (Ferreira et al., 2021); whereas *LACS4* family genes in tree peony respond to drought stress by mediating the synthesis of cutin and fatty acid precursors (Zhang et al., 2022). In addition, the plant ABCG family is widely involved in the transport of cuticular waxes, cuticular precursors and intracellular lipid signals, and plays an important role in drought adaptation (Rabeh et al., 2023).

In addition to membrane lipid system remodeling, the pentose-glucuronic acid interconversion pathway plays a central role in potato response to severe drought. This pathway mediates cell wall structural remodeling by providing nucleotide sugar precursors required for pectin and hemicellulose synthesis (Araujo et al., 2025; Mutanda et al., 2026). In this study, multiple key cell wall-remodeling genes in this pathway showed significant differential expression. Among them, the pectate lyase genes LOC102603970, LOC102605687 and LOC102602135 are involved in the regulation of pectin degradation; the pectin methylesterase (PME) family genes LOC102585483, LOC102591905, LOC102598327 and PME1 dominate the dynamic modification of cell wall pectin (Pecatelli et al., 2026). Drought-tolerant potato can coordinately regulate the expression patterns of PME isoforms and PME inhibitors (PMEIs), construct a mosaic pectin structure with alternating rigid and loose regions, maintain xylem structural integrity, reduce water loss through cell wall pores and inhibit cavitation expansion, and ultimately significantly improve plant drought tolerance (Jian et al., 2022; Zheng et al., 2022).

The phenylpropanoid biosynthesis pathway mediates adaptive regulation of secondary metabolism in potato under severe drought stress. The hub gene LOC102601955 encodes caffeic acid 3-O-methyltransferase (*COMT1*), a key methyltransferase for lignin monomer biosynthesis (Saluja et al., 2021). This gene is positively correlated with protective metabolites including terpenoids, steroidal glycosides and membrane lipids, while negatively regulating the accumulation of phenylpropanoid phenolamide derivatives. LOC102595175 encodes an acetyl-CoA benzyl-alcohol acetyltransferase-like protein belonging to the BAHD acyltransferase family, which also exerts a negative regulatory effect on phenolamide derivatives. Proteins of this family catalyze acyl-CoA-mediated secondary metabolic modifications. Previous studies have demonstrated that drought-inducible BAHD acyltransferase genes promote the accumulation of phenolic ester products, enhance plant mechanical tolerance, alleviate oxidative damage and optimize water retention capacity in potato and other crops (Zheng et al., 2022).

### MAPK signaling module-mediated regulatory network of transcriptional-metabolic resource trade-offs under drought stress

The MAPK signaling pathway can transmit drought stress signals from the extracellular environment to the nucleus, ultimately activating the expression of downstream stress-tolerance genes (Kumar et al., 2020). The WRKY transcription factor family, one of the largest transcription factor families in plants, plays a central role in mediating the transmission of MAPK signals to downstream metabolic pathways (Javed and Gao, 2023). Tang and Xia showed that WRKY transcription factors are key regulators of plant drought tolerance (Tang and Xia, 2025). In maize, the Zmhdz9 transcription factor enhances drought tolerance by regulating the accumulation of ABA and lignin (Li et al., 2024).

The WRKY family members LOC102587289 and LOC102588093 were significantly upregulated under both T1 and T2 treatments and were enriched in the MAPK signaling pathway and the plant–pathogen interaction pathway, suggesting that these two genes may serve as key regulatory nodes in response to specific stress conditions. The global correlation analysis in this study showed that gene sets associated with the MAPK and hormone signal transduction pathways were strongly positively correlated with lipids and steroid metabolites, but strongly negatively correlated with flavonoid and phenolic metabolites. This indicates that these two upstream signaling pathways may function as core hubs coordinating downstream multi-pathway metabolic reprogramming.

Lipids and steroid metabolites can contribute to plant drought adaptation by maintaining membrane stability as structural components of cell membranes, acting as precursors of signaling molecules involved in hormone biosynthesis such as jasmonic acid (JA), and forming hydrophobic cuticular barriers that reduce non-stomatal water loss (Samuels et al., 2008; Yang et al., 2023). The strong positive correlations between the MAPK and hormone signaling pathways and these two metabolite classes suggest that potato may preferentially activate a drought adaptation mode dominated by membrane repair and hormone-mediated signaling under different drought stress conditions.

The strong negative correlations between gene sets of the MAPK and hormone signal transduction pathways and flavonoid and phenolic metabolites indicate the presence of inverse regulation and resource competition between the expression of upstream signaling pathway genes and the accumulation of specific downstream metabolites (Gao et al., 2024; Zhou et al., 2025). This reflects that, in response to stress, potato relies on a precise transcriptional regulatory network to achieve dynamic trade-offs in metabolic resource allocation, rather than simply activating or inhibiting pathways in a linear manner.

## Conclusion

In this study, the major potato cultivar ‘Jizhangshu 12’, widely grown in arid and irrigated regions of North and Northwest China, was used as the experimental material. By integrating transcriptomic and widely targeted metabolomic approaches, the coordinated transcriptional–metabolic responses of potato leaves under moderate and severe drought stress were systematically analyzed. The transcriptome sequencing (RNA-seq approach) results were validated by RT-qPCR and showed good reliability.

The results showed that, as drought stress intensity increased, the number of differentially accumulated metabolites in potato leaves increased significantly, and the proportion of downregulated metabolites also rose markedly, whereas the total number of differentially expressed genes was higher under moderate drought stress. Functional enrichment and integrated multi-omics analyses indicated that phenylpropanoid–flavonoid biosynthesis, α-linolenic acid metabolism, glycerophospholipid metabolism, and MAPK and hormone signaling pathways were core regulatory modules involved in the drought response of potato. The *COMT1* gene LOC102601955, *F3’H* gene LOC102596126, ABCG transporter gene LOC102602737, and WRKY transcription factor gene LOC102587289 were identified as key candidate drought-tolerance genes.

Further correlation analysis showed that the upstream MAPK signaling pathway may positively regulate the accumulation of lipid and steroid metabolites while negatively regulating the biosynthesis of flavonoids, phenolics, and alkaloid secondary metabolites. The coordinated regulation of secondary metabolism differed markedly between moderate and severe drought stress. Under severe drought stress, the number of pathways involved in diverting carbon skeletons toward secondary defensive metabolites was significantly reduced, and the metabolic regulatory network became more concentrated.

Overall, this study elucidates distinct transcriptional–metabolic co-regulatory networks in potato under different drought stress conditions. The identified core drought-tolerance genes and characteristic metabolic markers provide important theoretical support and genetic resources for molecular breeding of drought-tolerant potato cultivars and for the development of water-saving cultivation systems in northern arid regions.

## Data Availability

The datasets presented in this study can be found in online repositories. The names of the repository/repositories and accession number(s) can be found below: https://www.ncbi.nlm.nih.gov/, PRJNA1494165.
