# Supplementary material for: Integrated multi-omics analysis reveals key genes and metabolic regulatory networks involved in the drought stress response of potato
Source: Front Plant Sci. 2026 Oct 6;17:1954199. doi: 10.3389/fpls.2026.1954199 (PMC13680202; doi:10.3389/fpls.2026.1954199)
Supplement: Supplementary file 1 [file SupplementaryFile1.docx]

**Supplementary Table S1. List of qRT-PCR primers**

| **Gene ID** | **Gene name / Putative function** | **Forward primer (5′→3′)** | | **Reverse primer (5′→3′)** | **Amplicon size (bp)** |
| --- | --- | --- | --- | --- | --- |
| LOC102577868 | Glyceraldehyde**-**3**-**phosphate dehydrogenase (GAPDH) — reference gene | GCTCCCATCACTTAACGG | ACCCTCTGATTCCTCCTTG | | 145 |
| LOC102587617 | Subtilisin**-**like protease SBT1.2 — proteolysis / stomatal development / stress response | GTGATTGCTCCAGGTGTCAAC | ATCCTCAGCCAGGCCACTA | | 72 |
| LOC102605888 | Chlorophyll a**-**b**-**binding protein 3C**-**like — photosynthesis / light harvesting | TTAAGAACCGTGAGTTGGAGG | AAGTGGATCAACAACCTCACC | | 155 |
| LOC102605534 | Beta‑galactosidase 10 — carbohydrate metabolism / cell wall | AGGCACTGCAATGCTTTTCG | TCCAGATGCACTGGCTTGAA | | 85 |
| LOC102585301 | Polygalacturonase‑like — cell wall pectin metabolism | GGCAAGGCCTAGCAATGGAT | TTTATGCCCGAGCTCTGCTG | | 141 |
| LOC102603846 | Pectinesterase 2 — cell wall modification / pectin metabolism | GATCAGCCACAGTTGGTGTTT | ATCGAAGGGCAACTGCTTGG | | 102 |
| LOC102600960 | MYB‑related protein 308**-**like — transcription factor / phenylpropanoid regulation | AGCAGCAGAAAGGTACAAGCA | CCCGTTCTTGAAAGCCCTGC | | 148 |
| LOC102587289 | Probable WRKY transcription factor 30 — MAPK signaling / plant‑pathogen interaction | GCACATTCTACTTCCCCTGCT | GCGGTGTACTGCTCTTTCTCT | | 158 |
| LOC102588093 | Probable WRKY transcription factor 26 (StWRKY9) — MAPK signaling / plant‑pathogen interaction | AGGCAAAGAGATGGAAGGGTG | TCAATGTCGCTTGTGGTTTGA | | 172 |
| LOC102579266 | Cysteine proteinase 3**-**like — proteolysis / stress response | TGCAGGGTACACCTCAAAACT | TTCCCTCCAGTCTTGCCTCT | | 92 |
| LOC102579608 | Glycerophosphodiester phosphodiesterase GDPD3**-**like — glycerophospholipid metabolism / membrane lipid remodeling | CAGACCGCCTATCGGGTTTT | ATGCCTTGTAGACCGCCTTC | | 119 |

**Supplementary Table S2. Statistics of RNA‑seq sequencing data**

| **Sample** | **Raw Reads** | **Clean Reads** | **Clean Base(G)** | **Error Rate(%)** | **Q20(%)** | **Q30(%)** | **GC Content(%)** |
| --- | --- | --- | --- | --- | --- | --- | --- |
| CK-1 | 53542406 | 52654600 | 7.9 | 0.03 | 98.11 | 94.24 | 43.23 |
| CK-2 | 53863056 | 52374846 | 7.86 | 0.02 | 98.25 | 94.54 | 43.04 |
| CK-3 | 56875408 | 55713250 | 8.36 | 0.02 | 98.23 | 94.49 | 43.21 |
| T1-1 | 55826696 | 54782968 | 8.22 | 0.02 | 98.21 | 94.42 | 42.99 |
| T1-2 | 58091976 | 56777482 | 8.52 | 0.03 | 98.12 | 94.23 | 42.88 |
| T1-3 | 61719798 | 60211700 | 9.03 | 0.02 | 98.23 | 94.48 | 42.85 |
| T2-1 | 50154708 | 48951068 | 7.34 | 0.02 | 98.35 | 94.84 | 42.97 |
| T2-2 | 48382654 | 47346850 | 7.1 | 0.02 | 98.39 | 94.89 | 43.08 |
| T2-3 | 46444988 | 45522552 | 6.83 | 0.02 | 98.42 | 94.98 | 43.07 |

Note: Raw reads: total number of reads from raw sequencing data; Clean reads: total number of reads after quality control; Clean bases: total data yield after quality control; Error rate (%): average base‑calling error rate of clean reads; Q20 (%), Q30 (%): percentage of bases with sequencing quality higher than 99% and 99.9%, respectively; GC content (%): percentage of total bases contributed by guanine (G) and cytosine (C) in clean reads.

**Supplementary Table S3.** **Key drought-responsive genes identified in the integrated analysis**

| **Gene ID** | **Gene/Enzyme Name** | **Pathway** | **log2FC** | **padj** | **Stress Level** |
| --- | --- | --- | --- | --- | --- |
| LOC102601955 | Caffeic acid 3-O-methyltransferase (COMT1) | Phenylpropanoid biosynthesis | Moderate: -1.144; Severe: -1.434 | Moderate: 1.49e-05; Severe: 1.47e-04 | Moderate & Severe |
| LOC102597674 | Chloroplast pyruvate kinase (PKG) | Pyruvate metabolism | -1.191 | 3.88e-03 | Moderate |
| LOC102601823 | Mitochondrial malate dehydrogenase (mMDH) | Pyruvate metabolism | -1.027 | 1.62e-06 | Moderate |
| LOC102597684 | Peroxisomal ascorbate peroxidase (APX3) | Ascorbate and aldarate metabolism | -1.021 | 3.53e-02 | Moderate |
| LOC102596126 | Flavonoid 3'-monooxygenase (F3'H) | Flavonoid biosynthesis | -1.2897 | 0.027 | Moderate |
| LOC102595103 | Cytosolic glutamine synthetase (GS1-1) | Biosynthesis of amino acids | -1.559 | 1.71e-09 | Moderate |
| LOC102603300 | NADP-dependent malic enzyme (NADP-ME) | Biosynthesis of amino acids | 2.009 | 1.05e-03 | Moderate |
| LOC102586126 | Phospholipase A1-IIγ | Glycerophospholipid metabolism | -1.250 | 5.49e-04 | Severe |
| LOC102578151 | Long-chain acyl-CoA synthetase 4 (LACS4) | Fatty acid metabolism | -2.379 | 1.65e-09 | Severe |
| LOC102590505 | Chloroplast 3-oxoacyl-ACP synthase I (KAS I) | Fatty acid metabolism | -1.158 | 9.71e-03 | Severe |
| LOC102602737 | ABC transporter G family member 11 (ABCG11) | ABC transporters | -1.677 | 5.88e-05 | Severe |
| LOC102603970 | Pectate lyase 8 | Pentose and glucuronate interconversions | -2.213 | 1.50e-04 | Severe |
| LOC102605687 | Pectate lyase 8 | Pentose and glucuronate interconversions | -1.713 | 1.20e-02 | Severe |
| LOC102602135 | Pectate lyase 5 | Pentose and glucuronate interconversions | -2.727 | 1.06e-02 | Severe |
| LOC102585483 | Pectin methylesterase (PME) | Pentose and glucuronate interconversions | -3.731 | 3.90e-04 | Severe |
| LOC102591905 | Pectin methylesterase (PME) | Pentose and glucuronate interconversions | -1.382 | 1.37e-03 | Severe |
| LOC102598327 | Pectin methylesterase (PME) | Pentose and glucuronate interconversions | -2.729 | 5.40e-06 | Severe |
| PME1 | Pectin methylesterase 1 (PME1) | Pentose and glucuronate interconversions | -2.824 | 1.16e-02 | Severe |
| LOC102595175 | Acetyl-CoA benzyl alcohol acetyltransferase-like protein | Phenylpropanoid biosynthesis | -2.559 | 1.50e-04 | Severe |
| LOC102587289 | WRKY transcription factor | MAPK signaling / Plant-pathogen interaction | Moderate: 1.983; Severe: 2.969 | Moderate: 2.45e-02; Severe: 1.74e-04 | Moderate & Severe |
| LOC102588093 | WRKY transcription factor | MAPK signaling / Plant-pathogen interaction | Severe: 2.520 | Severe: 2.97e-02 | Moderate & Severe |

**Supplementary Table S4. Representative differential metabolites identified in the integrated analysis**

| Metabolite Name | Category | Stress Level | Associated Gene(s) | Correlation | log2FoldChange | P-value |
| --- | --- | --- | --- | --- | --- | --- |
| Lysophosphatidylcholine 20:3 | Membrane lipid / Glycerophospholipid | Moderate | LOC102601955(COMT1), LOC102597684(APX3) | Positive | -1.373 | 1.867e-02 |
| Nardosinone | Terpenoid | Moderate & Severe | LOC102601955(COMT1) | Positive | Moderate: -1.071; Severe: -1.666 | Moderate: 3.262e-02; Severe: 1.125e-02 |
| Makisterone C | Terpenoid / Steroid | Moderate & Severe | LOC102601955(COMT1), LOC102597674(PKG) | Positive | Moderate: -1.207; Severe: -1.644 | Moderate: 5.788e-03; Severe: 2.848e-03 |
| Pingpeinone | Terpenoid / Secondary metabolite | Moderate | LOC102597674(PKG) | Positive | 1.177 | 4.668e-02 |
| 2-Propylmalic acid | Organic acid | Moderate | LOC102601823(mMDH) | Positive | -1.718 | 8.305e-03 |
| 3-Isopropylmalic acid | Organic acid | Moderate | LOC102601823(mMDH) | Positive | -1.718 | 8.305e-03 |
| Isoorientin | Flavonoid | Moderate | LOC102597684(APX3), LOC102596126(F3'H) | Negative | Induced | 3.418e-02 |
| N-Acetyl-L-glutamic acid | Amino acid / Osmolyte | Moderate | LOC102595103(GS1-1) positive; LOC102603300(NADP-ME) negative | Bidirectional | -1.352 | 3.366e-03 |
| 15(R)-Hydroxy-linoleic acid | Fatty acid / Hydroxy fatty acid | Severe | LOC102578151(LACS4) | Positive | -1.829 | 8.482e-03 |
| 5-Pregnenetriol-20-O-glucoside | Steroid glycoside | Severe | LOC102601955(COMT1) | Positive | -1.703 | 1.057e-02 |
| Caffeoyl agmatine | Phenylpropanoid phenolamide | Severe | LOC102601955(COMT1), LOC102595175 | Negative | 2.346 | 1.529e-02 |
| Feruloyl spermidine | Phenylpropanoid phenolamide | Severe | LOC102601955(COMT1), LOC102595175 | Negative | 1.414 | 1.492e-02 |
| N-Feruloyl homoagmatine | Phenylpropanoid phenolamide | Severe | LOC102601955(COMT1), LOC102595175 | Negative | 1.864 | 2.575e-02 |
| N-Feruloyl methylagmatine | Phenylpropanoid phenolamide | Severe | LOC102601955(COMT1), LOC102595175 | Negative | 2.310 | 1.719e-02 |
| Quercetin-3-O-arabinoside | Flavonoid | Moderate | — | Drought-induced accumulation | 2.164 | 3.110e-01 |
| Gossypetin-3-O-rutinoside | Flavonoid | Severe | — | Drought-induced accumulation | 2.440 | 1.847e-01 |

Note: Induced represents metabolites not detected in control samples and only present under drought stress.

**Supplementary Figure S1.** Permutation test (200 permutations) for OPLS**-**DA models. (A) Permutation test for the OPLS**-**DA model of T1 vs CK. (B) Permutation test for OPLS**-**DA model of T2 vs CK. The intercept of Q_2_ indicates no over**-**fitting of the model.

**Supplementary Figure S2.** Pearson correlation heatmap of transcriptome samples. Pair**-**wise correlation among all biological replicates demonstrates high reproducibility within each treatment group.

**Supplementary Figure S3.** Association network between core differentially expressed genes and differentially accumulated metabolites under drought stress. (A) T1 vs CK. (B) T2 vs CK.
